# Prevalence of Intestinal Parasitic Infestation among Public School Children of a Community

**DOI:** 10.31729/jnma.4892

**Published:** 2020-05

**Authors:** Manisha Sharma, Jyotshna Sapkota, Beena Jha, Bhavesh Mishra, Chandra Prakash Bhatt

**Affiliations:** 1 Department of Microbiology, Kathmandu Medical College and Teaching Hospital, Sinamangal, Kathmandu, Nepal.

**Keywords:** *intestinal diseases*, *parasitic*, *prevalence*, *schools*

## Abstract

**Introduction:**

Intestinal parasitic infestation is one of the major health problems in developing countries like Nepal. This study was done to determine the prevalence rate of intestinal parasitic infestation among school children in Duwakot VDC, Bhaktapur, Nepal.

**Methods:**

A descriptive cross-sectional study was done in 194 public school children of Duwakot village development committee from August to October, 2019. Ethical clearance was obtained from the Institutional Review Committee (reference no. 1207201915). Simple random sampling was done. One hundred and ninety-four public school children individuals of 6 to 14 years of age were enrolled. Collected stools were examined for the presence of parasites macroscopically and microscopically. Microscopic examination was carried out by direct wet mount using normal saline (0.9%) and Lugol’s iodine (0.5%) mount. The data obtained were computed and analyzed using Statistical Package for the Social Sciences version 16.0.

**Results:**

A total of 194 stool samples were collected from school children and examined. The prevalence of intestinal parasitosis was 26 (13.40%). The commonest organism was *Giardia lamblia* in 22 (11.34%) cases. Among helminthic infection, 2 (1.03%) cases each were infected by *Hymenolepis nana* and Hookworm respectively.

**Conclusions:**

The prevalence rate of intestinal parasite infestation in Nepal shows considerable decline in recent years. However, more effort is required by public health resources to minimize the problem further.

## INTRODUCTION

Significant morbidity and mortality are associated with infectious diseases in Nepal, with intestinal parasitosis (protozoan and helminthic infections) contributing as one of the major cause.^1^

World Health Organization (WHO) estimates that more than 880 million children are in need of treatment for soil-transmitted helminth infections.^2^ The prevalence rate of these infections is impacted by factors like mode of transmission, local parasite burden, proper handling of human and animal waste, and availability of clean drinking water for inhabitants.^3^ WHO has set Global target to eliminate soil transmitted helminthic infections by 2020.^2^ In Nepal, programs like public health education, providing anti helminthic chemotherapy for children periodically is being done. The success of these measures can be in part be discerned by the effect on health status of the population.

Therefore, this study was done to determine the prevalence of intestinal parasitic infestation in Public School children in Duwakot VDC, Bhaktapur, Nepal.

## METHODS

A descriptive cross-sectional study was done in 194 public school children of Duwakot VDC from August to October, 2019. Ethical approval was taken from Institutional Review Committee (IRC), Ref no. 1207201915, KMCTH. Written consent was taken from the parents of the participants. Demographical data like age, gender, caste were noted. The patients were provided with dry, screw capped, wide mouthed container for collection of samples. Stool sample was collected, labeled and transported to laboratory in Microbiology department in KMC Duwakot for processing. Collected stools were examined for the presence of parasites macroscopically and microscopically. Microscopic examination was carried out by direct wet mount using normal saline (0.9%) and Lugol's iodine (0.5%) mount. Simple random sampling was done.

The sample size (n) was calculated as follows,

n = Z^2^ × p × q/e^2^

= (1.96)^2^ × 0.5 × 0.5/(0.06)^2^

= 266.78

Where,

Z= 1.96 for 95% confidence interval
p= prevalence = 50%
q= 1-p
e= margin of error = 7%
N = no. of total students in the public schools i.e. study
site = 550

For finite population,

N_0_ = n/ 1 +(n-1/N)
= 266.78/1 +(266.78-1/550)
= 266.78/1.483
= 179.86

A total of 194 children aged 6-14 years attending public school in Duwakot VDC were included in the study statistical analysis was performed using Statistical Package for the Social Sciences 16.0 Version.

## RESULTS

**Figure 1:**
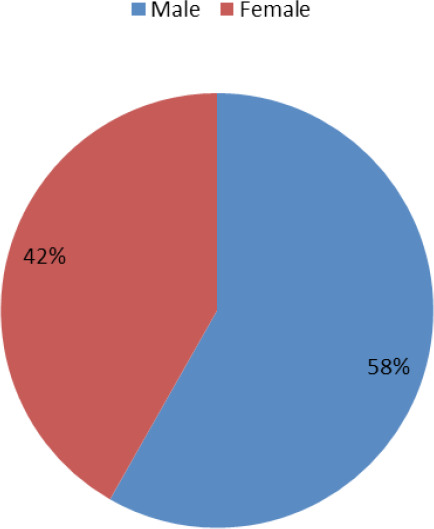
Distribution of male and female participants.

A total of 194 stool samples were collected from school children and examined. Among the participants, 113 (58.24%) were male and 81 (41.75%) were female (Figure 1).

**Figure 2:**
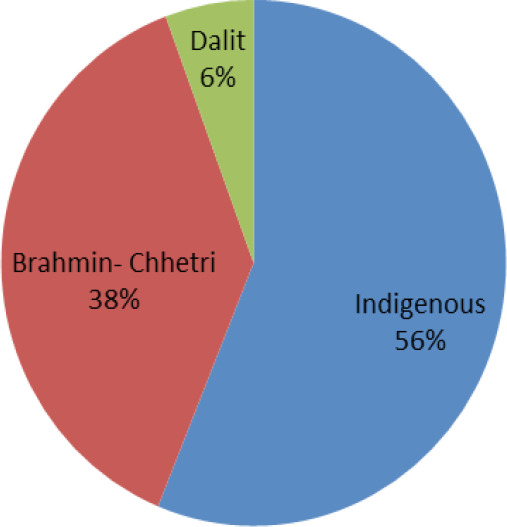
Distribution pattern of participants according to caste.

The highest number of participants belonged to Aadibasi-Janjati 109 (56.18%) followed by Brahmin-Chhetri 74 (38.14%) and Dalit 11 (5.67%) (Figure 2).

The prevalence of intestinal parasitosis was 26 (13.40%). The commonest organism was Giardia lamblia 22 (11.34%). Among helminths, 2 (1.03%) cases each were infected by by Hymenolepis nana and Hookworm respectively (Table 1).

**Table 1:** Distrubution of parasitic inestation among the participants.

Parasites	n (%)
Giardia lamblia	22 (11.34)
Hymenolepis nana	2 (1.03)
Hookworm	2 (1.03)
Total	26 (13.40)

## DISCUSSION

The burden of intestinal parasitic infestation (IPI) is a significant health concern esp. among schoolchildren in Nepal.^4^

The prevalence of intestinal parasitosis in our study was 13.40%. Various studies from Nepal have reported the prevalence of IPIs in school children to be 19.9%, 16.7% and 22.5%.^4-6^ The relatively low prevalence in our study may be in part due to comparatively recently introduced public health measures like periodic deworming, awareness messages circulated in mass media.

The protozoan parasite Giardia lamblia was the commonest parasite isolated 22 (11.34%). Similar rate (11.45%, 13.6%) was reported by Tandukar et al. and Narayan et al respectively.^5,6^ Other studies from Nepal have reported the prevalence to be (26.0%, 30.5%).^7,8^ The higher rate of protozoan infestation in one study may be due to contamination of drinking water by the cysts and its capacity to resist normal level of chlorine treatment in drinking water. We noted lower prevalence of helminthic infection compared to protozoal infection in our study. Periodic deworming by providing antihelminthic drug to school children may have played a part in lowering the prevalence rate. Moreover, Nepal also declared all 77 districts as open defecation free area on 30/9/2019. A meta analysis from Nepal reported the burden of helminthes to be higher in rural areas, whereas in urban areas, the burden of protozoa was higher.^9^Our study site was in Bhaktapur, adjacent to Kathmandu.

The prevalence of parasitic infections in our study was statistically independent of gender, ethnicity. Similarly Pradhan et al. and Mall B et al. reported the prevalence to be statistically independent of gender.^10,11^ However, in another study by Agarwal et al, positive rate was higher in Dalit and Aadibasi-Janjati than Brahman-Chhetri.^7^

In a meta analysis from Nepal, significant decrease in prevalence of intestinal parasite infection was reported (20%-recent 5 years to 61%- late 1990s).^9^The source of infestation is the carrier or the infested person and in most cases the mode of transmission is faeco-oral.^12^ Improving sanitation, hygiene and overall health of children in Nepal might be contributing on reducing the burden of intestinal parasites in Nepal.^9^

The examination of consecutive stool samples, use of concentration method could have further improved the yield of parasites.

## CONCLUSIONS

Though exhibiting decreasing trend. Intestinal parasitosis is still an important public health concern perpetuated by lack of education, poverty, inadequate access to clean drinking water, improper sewage disposal. Effective public health measures along with periodic screening and treatment is warranted for decreasing the burden of disease in Nepal.
